# Epidemiology of floods in sub-Saharan Africa: a systematic review of health outcomes

**DOI:** 10.1186/s12889-022-12584-4

**Published:** 2022-02-10

**Authors:** Friederike Suhr, Janina Isabel Steinert

**Affiliations:** 1 School of Social Sciences and Technology, Technical University of Munich, Richard-Wagner Str. 1, 80333 Munich, Germany; 2 Department of Social Policy and Intervention, University of Oxford, Oxford, UK

**Keywords:** Systematic review, Sub-Saharan Africa, Natural disasters, Vector-borne diseases, Zoonotic diseases, Water-borne diseases

## Abstract

**Background:**

Floods have affected 2.3 billion people worldwide in the last 20 years, and are associated with a wide range of negative health outcomes. Climate change is projected to increase the number of people exposed to floods due to more variable precipitation and rising sea levels. Vulnerability to floods is highly dependent on economic wellbeing and other societal factors. Therefore, this systematic review synthesizes the evidence on health effects of flood exposure among the population of sub-Saharan Africa.

**Methods:**

We systematically searched two databases, Web of Science and PubMed, to find published articles. We included studies that (1) were published in English from 2010 onwards, (2) presented associations between flood exposure and health indicators, (3) focused on sub-Saharan Africa, and (4) relied on a controlled study design, such as cohort studies, case-control studies, cross-sectional studies, or quasi-experimental approaches with a suitable comparator, for instance individuals who were not exposed to or affected by floods or individuals prior to experiencing a flood.

**Results:**

Out of 2306 screened records, ten studies met our eligibility criteria. We included studies that reported the impact of floods on water-borne diseases (*n* = 1), vector-borne diseases (*n* = 8) and zoonotic diseases (*n* = 1). Five of the ten studies assessed the connection between flood exposure and malaria. One of these five evaluated the impact of flood exposure on malaria co-infections. The five non-malaria studies focused on cholera, scabies, taeniasis, Rhodesian sleeping sickness, alphaviruses and flaviviruses. Nine of the ten studies reported significant increases in disease susceptibility after flood exposure.

**Conclusion:**

The majority of included studies of the aftermath of floods pointed to an increased risk of infection with cholera, scabies, taeniasis, Rhodesian sleeping sickness, malaria, alphaviruses and flaviviruses. However, long-term health effects, specifically on mental health, non-communicable diseases and pregnancy, remain understudied. Further research is urgently needed to improve our understanding of the health risks associated with floods, which will inform public policies to prevent and reduce flood-related health risks.

**Supplementary Information:**

The online version contains supplementary material available at 10.1186/s12889-022-12584-4.

## Background

Flooding has been the most common type of natural disaster in the last 20 years, accounting for 47% of all recorded natural disasters and affecting 2.3 billion people worldwide [1]. Current data suggests that floods are the deadliest of all forms of natural disaster, responsible for 43.5% of all deaths from natural disasters in 2019 [2]. Vulnerability to the health consequences of natural disasters is highly dependent on the adaptive capacities of countries or regions. For instance, low-income countries experience more than three quarters of the global mortality burden caused by natural disasters [3]. In this regard, sub-Saharan Africa (SSA) exhibits very challenging and concerning preconditions. The number of people living in extreme poverty, defined as living on $1.90 or less per day, continues to rise in SSA while declining in all other regions of the world [4]. Twenty-seven out of the 28 poorest countries in the world are located in SSA and it is estimated that by 2030, SSA will be home to nine in ten of the world’s extreme poor [4, 5]. Compounding the economic challenges is the rapid urbanization of sub-Saharan African cities, which is accompanied by the expansion of informal settlements and unplanned water and sanitation infrastructure [6].

While SSA has made progress overall in reducing its disease burden, most sub-Saharan countries are facing a double burden of disease. Communicable diseases, such as HIV and malaria, make up much of the region’s disease burden, but SSA is experiencing a rapid shift towards increasing predominance of non-communicable diseases [7]. This epidemiological transition alone challenges health care systems in SSA, which are often under-resourced and fragile [7]. Consequently, pre-existing health challenges are exacerbated under flood conditions. Floods may challenge ecological determinants of good health, including safe drinking water, food security, and secure shelter [8–10]. For example, damaged or destroyed water and sanitation infrastructure may lead to drinking water contamination from sewage, agricultural waste, industrial waste or chemicals [11]. This leads to water scarcity and increased competition for water, and is associated with the spread of water-borne diseases such as cholera and typhoid [12–15]. Flooding and polluted water resources can also lead to soil contamination, which can damage crops and disrupt food supplies [16, 17]. Depending on the reliance on domestic food production, the effect of flooding on food security and population nutrition can be severe. It is alarming that the occurrence of droughts increased nearly threefold between 2010 and 2019, relative to the period 1970 – 1979, but more so that in the same period, the frequency of floods increased nearly tenfold [18]. Many sub-Saharan African countries lack climate adaptation strategies and policies to address the rising frequency of natural disasters [19]. There is an urgent need to develop plans to mitigate the impact of floods on health. The failure to prepare raises real concerns about the disease burden in SSA in the future. Insufficient financial capacity at the individual and country level constrains the ability to build flood resilience. Risk factors such as the reliance on unimproved drinking water, open-defecation practices, and communities living in close proximity to water bodies - combined with rapid urbanization, population growth, environmental determinants and the current disease burden - further exacerbate sub-Saharan Africa’s vulnerability to floods [6, 18, 20–24].

In this context, our systematic review examines how the exposure to floods affects any human-health related outcome in SSA. We aim to collate the quantitative evidence on the impact of flood exposure for population health in SSA and identify gaps in the literature. This synthesis of evidence includes quantitative studies with a controlled design, such as cohort studies, case-control studies, cross-sectional studies, or quasi-experimental approaches with a suitable comparator. An example of a suitable comparator is individuals who were not exposed to or affected by a flood, or individuals prior to experiencing a flood.

Previous literature reviews have concentrated on exploring the link between individual diseases and floods on a national or regional level [25, 26]. However, their results are limited to specific diseases and therefore cannot be used to evaluate broader impacts. Other systematic reviews focus on the combined impact of several natural disasters on human health [27–29]. Two systematic reviews specifically cover the health impacts of floods globally [30, 31]. Although, Alderman’s and colleagues (2012) systematic review did not include any study located in SSA, it was not restricted to a specific geographic region and thus provides an overview of the health effects of flood exposure between 2004 and September of 2011 [31]. An earlier review by Ahern and colleagues (2005) presents evidence on three floods in SSA and covers the time period up to 2004 [30]. Their evidence suggests that flood exposure in SSA is associated with an increasing incidence of malaria, diarrheal disease and polio [30]. Our systematic review builds on this and focuses explicitly on a more recent time period (2010 until today). Considering that incidence rates of malaria and polio have declined since the 1990s, our review contributes to the current literature by providing a comprehensive, up-to-date investigation of the health effects of flood exposure in SSA, and investigating its potential impact on other diseases [32, 33].

## Methods

### Design

To locate, summarize and evaluate the best available evidence on the health effects of floods in SSA, we conducted a thorough systematic review of quantitative studies following PRISMA guidelines [34]. Details of the protocol for this systematic review were registered on PROSPERO [35].

### Eligibility criteria

We included observational studies in the review if they reported on any physical or mental health effect of flood exposure (including mortality, morbidity, worsening/improving of conditions, injuries, etc.) and had a controlled research design (including cohort studies, case-control studies, cross-sectional studies, or quasi-experimental approaches with a suitable comparator, for instance individuals who were not exposed to or affected by floods or individuals prior to experiencing a flood). We did not apply any restrictions with regard to the use of primary and secondary data. The inclusion criteria required that studies were carried out in SSA, as defined by the United Nations, and were published in English in 2010 and later [36]. We did not apply any age or gender restrictions.

### Information sources and search strategy

We searched Web of Science (Web of Science Core Collection, SciELO Citation Index, Medline) and PubMed. The search strategy was developed and tested in an iterative process to ensure completeness, relevance, and maintain precision. It included three components, which refer to (i) the exposure to floods, (ii) the location, and (iii) health outcomes. While the parts of the search string that referred to the exposure to floods and the location were precise because they contained only synonyms, abbreviations or other spelling options, the part that related to health outcomes was intentionally kept broad and comprised generic terms, such as health or mortality. The complete search strategy is outlined in the supplementary file 1.

We also conducted a hand search of the reference lists of related systematic reviews. This yielded five additional records.

### Study selection

**Fig. 1:**
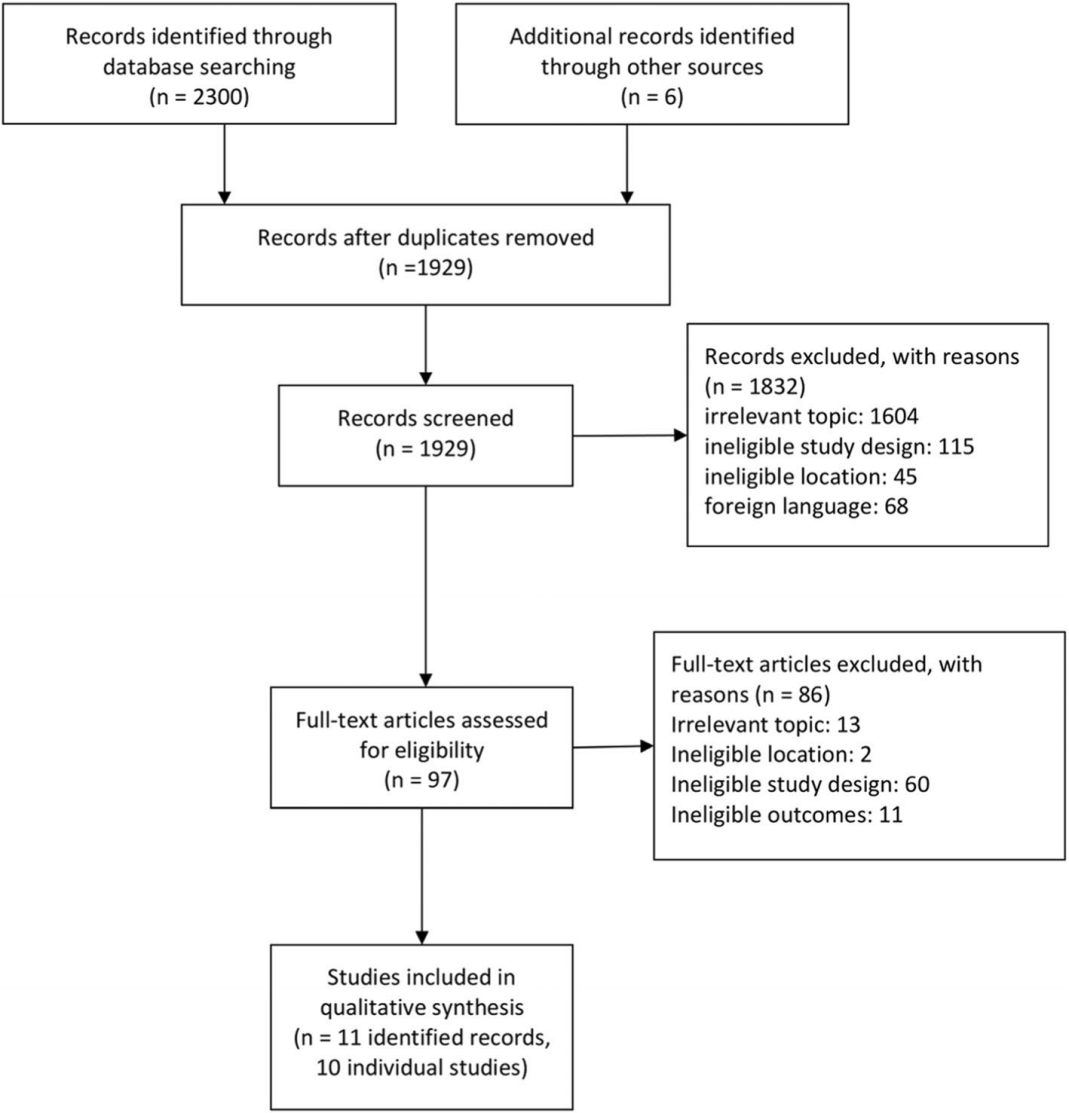
Flow chart of study selection adapted from Moher and colleagues (2009) [34]

We uploaded 2306 records into the online software Rayyan. We checked and removed duplicates, leaving 1929 records to be screened by title and abstract. 1832 records were excluded because they did not meet the study eligibility criteria. Reasons for exclusion were irrelevant topic (*n* = 1604), ineligible study design (*n* = 115), ineligible location (*n* = 45) or foreign language (*n* = 68). We assessed the remaining articles (*n* = 97) for eligibility based on their full text. Most were excluded due to ineligible study design (*n* = 60), irrelevant topic (*n* = 13), ineligible outcome (*n* = 11) or ineligible location (*n* = 2). Uncertainties about inclusion or exclusion of studies were discussed with a second independent investigator (JIS). The overall study identification process and the specific stages of the screening process are depicted in the flow chart in Fig. 1.

### Data extraction

Data of the studies that met the eligibility criteria was extracted using the following categories: author and year, country and year (if available) of flood, study design, data types (clinical data or survey-based data), study participants, sample size, exposure, outcome, effect measures and mechanisms of disease transmission. We grouped included studies into disease types, namely water-borne diseases, vector-borne-diseases, and zoonotic diseases. Data was entered into a table in Microsoft Excel. To ensure that the extracted data, was accurate, a second independent investigator (JIS) checked the data extraction table.

## Results

### Study characteristics

We reduced the 2306 identified papers down to eleven records referring to ten individual studies for the qualitative synthesis (see Fig. 1). Two studies by Mboera and colleagues were grouped together because their study published in 2011 builds on data retrieved from the study published in 2010 [37, 38].

**Fig. 2:**
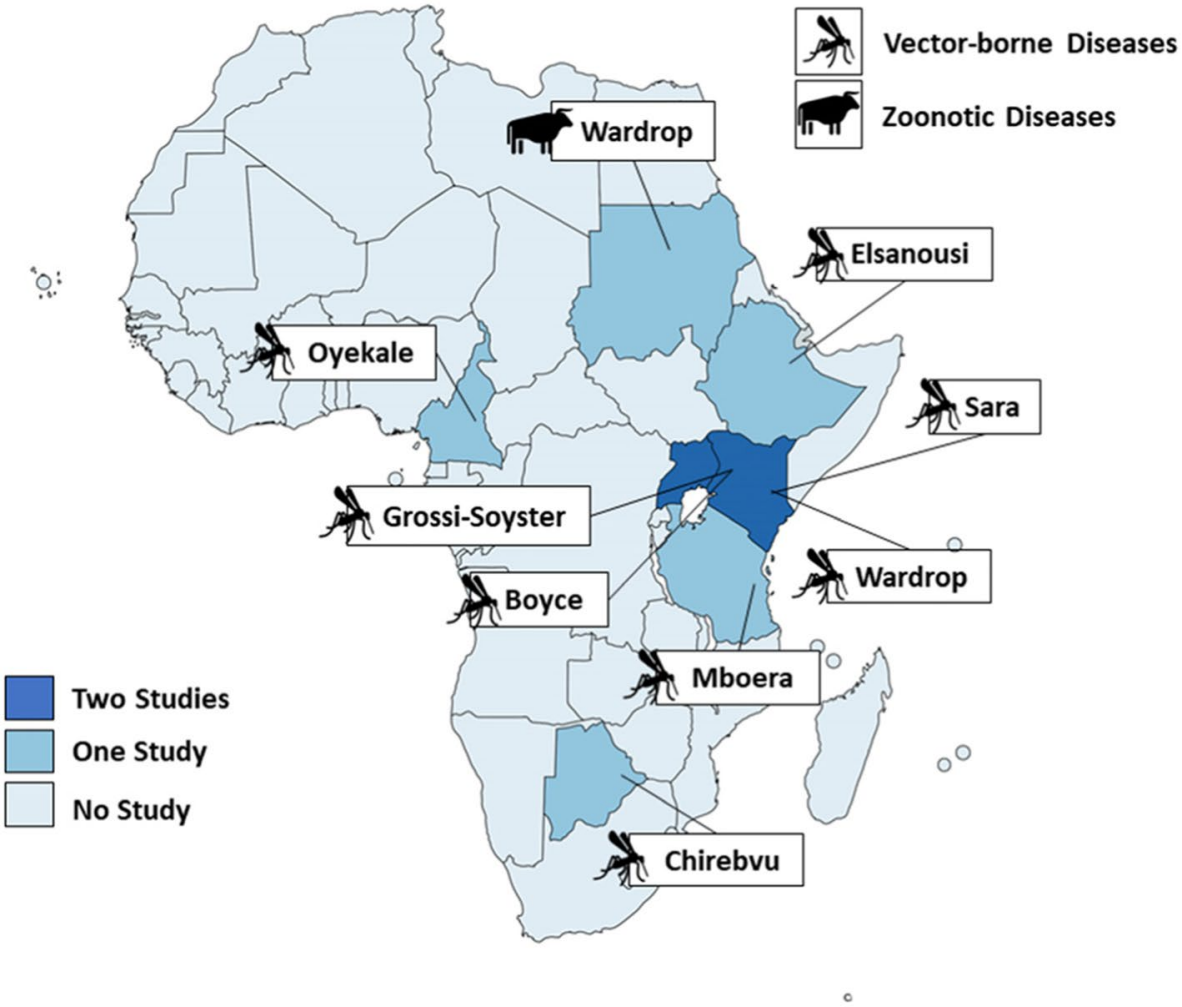
Geographic distribution of included studies – Note: For better visual representation, the Rieckmann and colleagues (2018) study is excluded from the figure [39]

The identified studies were carried out in Uganda (*n* = 2), Botswana (*n* = 1), Sudan (*n* = 1), Kenya (*n* = 2), Ethiopia (*n* = 1), Tanzania (*n* = 1) and Cameroon (*n* = 1) (see Fig. 2). One additional study by Rieckmann and colleagues (2018) comprised data from 40 sub-Saharan African countries [39]. Of the studies that refer to a single country, four of the countries (Sudan, Kenya, Tanzania and Cameroon) have a coastline and three are landlocked (Uganda, Botswana, Ethiopia). Five of the ten included studies assessed the impact of floods exclusively on human health in rural areas. The remaining studies evaluated the impacts on both rural and urban populations combined or did not further define the study population and setting.

There were two case-control studies, four cross-sectional studies and three longitudinal studies. Another study used a quasi-experimental design. Eight of the studies concentrated on vector-borne diseases, one on water-borne diseases and one on zoonotic diseases. The total sample size of the papers included in the systematic reviews is 40,453 individuals and disease cases, and 276 disease outbreaks from the Rieckmann and colleagues (2018) study [38]. Using the Emergency Events Database (EM-DAT), we estimate that approximately 40 million people have been affected by floods in SSA between 2010 and 2020 [40]. Following the EM-DAT’s definition of the total number of affected people, the estimate represents the sum of injured, affected, and homeless people after flood exposure [40]. As stated by EM-DAT, the estimates are still prone to underreporting and may be impaired due to different reporting standards [40].

More detailed information on the studies is provided in Table 1.

**Table 1:** Summary of included studies

Study	Country and Year of Flood^a^	Study Design	Data Type	Study Participants	Sample Size	Outcome	Effect Measures	Mechanisms of Disease Transmission
**Water-borne Diseases**								
Rieckmann et al., 2018 [39]	40 Sub-Saharan African countries^b^	Register-based, country-level longitudinal study of cholera outbreaks and flood events in sub-Saharan Africa between 1990 and 2010.	Disease surveillance data	Not applicable	276 cholera outbreaks	Cholera outbreaks	Incidence rates of cholera outbreaks were elevated during flood periods when compared to periods not affected by floods (IRR = 144; 95% CI: 101–208, *p*-value not discussed).	− Overflowing of the sanitation systems − Contamination of the environment and water sources − Displacement and influx of aid workers facilitates transmission of diseases − Overcrowding, which exacerbates hygiene and sanitation concerns
**Vector-borne Diseases**								
Oyekale, 2015 [41]	Cameroon	Cross-sectional study of 2011 Demographic and Health Survey (DHS) data. Clinical malaria cases and households dwelling characteristics, such as living in a flood-prone area, were analysed.	Clinical data and survey-based data.	Children, aged 6 to 59 months.	6623 children	Malaria infections	Children that resided in flood-prone areas compared to those who do not, had a 8.9 percentage points lower likelihood of a malaria infection (p-value < 0.01).	Not applicable
Mboera et al., 2011, Mboera et al., 2010 [37, 38]	Tanzania	Cross-sectional study. Clinical malaria cases in flooded and non-flooded ecosystems were investigated in 2005.	Clinical data	Schoolchildren in classes 1 to 4	578 schoolchildren	Malaria infections	The prevalence of plasmodium falciparum was significantly higher in a flooding rice irrigation environment than in a non-flooded sugarcane farming environment (OR = 10.14; CI: 4.58 - 22.42, p-value < 0.05).	Not applicable
Elsanousi et al., 2018 [42]	Sudan, 2013	Observational retrospective study of malaria data sets between 2011 and 2013. Comparison of data sets during the year of flooding (2013) with those of corresponding non-flood years (2011, 2012).	Clinical data	Children, adolescents and adults.	2011: 5069 malaria cases, 2012: 5549 malaria cases, 2013: 7262 malaria cases	Malaria infections	People exposed to floods in 2013 had a significantly higher slide positivity rate (%) than people not exposed to floods in 2011 (SPR = 2.39%; 95% CI: 2.27-2.51, p-value < 0.0001)	− Increased growth of Anopheles mosquito population through formation of new breeding sites and favourable conditions for mosquito development and survival. − Displacement, damage to private houses, and destruction of the infrastructure, may decrease the reduces accessibility of healthcare services.
Boyce et al., 2016 [43]	Uganda, 2013	Quasi-experimental design. Difference-in-difference approach to investigate the causal relationship between laboratory- confirmed malaria cases and different environmental factors in a pre- and a post-flood period.	Clinical data	Children, adolescents and adults.	7596 individuals	Malaria infections	The likelihood of receiving a positive test result was significantly higher in the post-flood period than in the pre-flood period (ARR = 1.47; 95% CI: 1.36-1.58, p-value < 0.001). The presence of a flood-affected river near the studied villages was associated with a significantly higher test positivity rate compared to villages farther from a river (ARR = 1.30; 95% CI: 1.16–1.46, *p*-value < 0.001).	− Creation of stagnant pools as ideal breeding habitats for the Anopheles mosquito
Chirebvu et al., 2016 [44]	Botswana	5-year retrospective time series analysis of clinical malaria cases and climate variables between flood and non-flood periods.	Clinical data	Children, adolescents and adults.	Not applicable.	Malaria infections	At a lag period of six month, the incidence of clinical malaria cases correlates most strongly with flood extent (ρ = 0.467, p-value < 0.05). When setting the lag period to zero months, the incidence of clinical malaria cases is most strongly associated with flood discharge (ρ = 0.396, p-value < 0.05).	− Emergence of suitable breeding habitats and thus influence growth of the Anopheles mosquito population.
Sara et al., 2018 [45]	Ethiopia	Unmatched case-control study (1:2 ratio). Analysis of scabies infections and individuals dwelling characteristics, such as home being affected by flooding.	Clinical data and survey-based data.	Individuals, aged 8 months to 70 years for the line-listed scabies cases. Individuals, aged 3 months to 65 years for the case-control analysis.	4532 line-listed scabies cases. 55 scabies cases and 110 controls for the case-control analysis.	Scabies infections	The odds of a scabies infection were approximately 22 times higher among people who lived in homes affected by flooding compared to people not affected by flooding (aOR = 22.32; 95% CI: 8.46–58.90, p-value < 0.0001).	− Displacement, overcrowding, and worsened personal hygiene
Grossi-Soyster et al., 2017 [46]	Kenya	Cross-sectional study. Analysis of links between serological samples and demographic data, information on lifestyle, previous state of health and recent experience of village flooding.	Clinical data and survey-based data.	Children and adults, aged 5-75.	250 children, 250 adults	Alphaviruses and flaviviruses seroprevalence	Recent experience of village flooding significantly increased the likelihood of alpha- or flavivirus infection (OR = 2.49; 95% CI: 1.31–4.73, p-value < 0.005).	− Emergence of potential environments for mosquito breeding.
Wardrop et al., 2013 [47]	Uganda	Matched case-control study design with 1:1 matching based on age group. Correlation between distribution of Rhodesian sleeping sickness and environmental factors, such as residence in a flooded environment, is investigated.	Clinical data	Children, adolescents and adults.	233 Rhodesian sleeping sickness cases and 233 controls.	Rhodesian sleeping sickness infections	Higher proportions of seasonally flooded grassland significantly increased the likelihood of Rhodesian sleeping sickness (OR = 1.18; 95% CI: 1.04-1.33, p-value = 0.01).	− Emergence of more favourable habitat for tsetse survival and reproduction.
**Zoonotic Diseases**								
Wardrop et al., 2015 [48]	Kenya	Cross-sectional study. Clinical laboratory diagnostics are used to determine the presence of a taeniasis infection and are linked to survey-based and environmental data on flooding.	Clinical data and survey-based data.	Individuals older than five years and not in the third trimester of pregnancy.	416 households, comprising 2113 individuals	Taeniasis infections	The presence of the antigen in the study population is significantly higher within a 1 km distance to flooded agricultural land and flooded grassland (OR = 1.09; 95% CI: 1.01-1.17, p-value = 0.03).	− Surface moisture and humidity increase likelihood of survival of Taenia spp. eggs − Floodwaters transport the *Taenia* spp. eggs to other areas

^a^ if applicable

^b^ Angola, Burundi, Djibouti, Eritrea, Ethiopia, Kenya, Malawi, Mozambique, Rwanda, Somalia, Tanzania, Uganda, Zambia, Cameroon, Central African Republic, Chad, Congo, Democratic Republic of Congo, Equatorial Guinea, Gabon, Botswana, Lesotho, Namibia, South Africa, Swaziland, Zimbabwe, Benin, Burkina Faso, Cote d’Ivoire, Gambia, Ghana, Guinea, Guinea Bissau, Liberia, Mali, Mauritania, Niger, Nigeria, Senegal, Sierra Leone, Togo

### Summary of evidence

Cholera was the only identified water-borne disease associated with the exposure to floods in this systematic review. Rieckmann and colleagues (2018) found an association between extraordinarily high incidence rates of cholera outbreaks during floods compared with flood-free periods (Incidence Rate Ratio (IRR) = 144; 95% CI: 101–208) [39]. The study presents evidence from 40 sub-Saharan African countries, which are listed in Table 1.

A home being affected by flooding was one associated risk factor for scabies infestation in the Sara and colleagues (2018) study, which was conducted in Ethiopia (adjusted OR (aOR) = 22.32; 95% CI: 8.46–58.90, *p*-value < 0.0001) [45].

The relationship between flooding and Rhodesian sleeping sickness is rather complex and depends on the year of observation and the statistical method used [47]. The results of the univariate analysis demonstrated lower odds of Rhodesian sleeping sickness infection in Ugandan villages that were surrounded by a higher proportion of seasonally flooded grassland [47]. However, after the first year the relationship reversed, and multivariate analysis indicated that higher proportions of seasonally flooded grassland resulted in an increased likelihood of the occurrence of Rhodesian sleeping sickness (OR = 1.18; 95% CI: 1.04-1.33, *p*-value   =  0.01) [47].

Alphavirus or flavivirus infections were found to be significantly correlated with a recent experience of village flooding (OR = 2.49; 95% CI: 1.31–4.73, p-value < 0.005) [46].

Half of the studies reported effects of flood exposure on malaria infections. A cross-sectional study was conducted in Tanzania to investigate the relationship between the prevalence of the pathogen responsible for malaria and different ecosystems [38]. The study by Mboera and colleagues (2011) found that the prevalence of the pathogen responsible for malaria was twice as high in flooding rice irrigation environments than in non-flooding irrigation environments (OR = 10.14; CI: 4.58 – 22.42, *p*-value < 0.05) [38]. A similar pattern was identified for coinfections with malaria and parasitic worms [42]. Elsanousi and colleagues (2018) found that Sudanese people exposed to floods in a specific year had a higher likelihood of a malaria infection than people not exposed to floods (Slide Positivity Rate^1^ (SPR) = 2.39%; 95% CI: 2.27-2.51, *p*-value < 0.0001) [42]. These findings are supported by Boyce and colleagues’ (2016) study, which was conducted in Uganda [43]. The authors found that the likelihood of receiving a positive test result for malaria infection was higher in the post-flood period than in the pre-flood period (Adjusted Rate Ratio (ARR) = 1.47; 95% CI: 1.36-1.58, *p*-value < 0.001) [43]. Additionally, the presence of a flood-affected river near the studied villages in the post-flood period was associated with a significantly higher positive test rate for malaria infection (ARR = 1.30; 95% CI: 1.16–1.46, *p*-value < 0.001) [43]. Chirebvu and colleagues (2016) discovered a more complex relationship between an infection with malaria and the exposure to floods in their 5-year retrospective analysis conducted in Botswana [44]. The authors investigated the transmission patterns of malaria and their relationship with flood extent (km^2^) and flood discharge (mm^3^/month). Flood extent is defined as the area of land which has been flooded, measured in km^2^. Flood discharge is defined as the volume of water in cubic millimetres that passes through a cross section per month. At a lag period of six months, the incidence of clinical malaria cases correlated most strongly with flood extent (Pearson’s ρ = 0.467, *p*-value < 0.05) [44]. When setting the lag period to zero month, the incidence of clinical malaria cases was most strongly associated with flood discharge (ρ = 0.396, *p*-value < 0.05)^2^ [44]. Chirebvu and colleagues (2016) interpret this as suggesting that transmission patterns of malaria coincide with receding floods [44].

Oyekale (2015) predicted marginal effects based on a probit regression model and reported an 8.9 percentage point reduced likelihood of a malaria infection among children who resided in flood-prone areas in Cameroon (p-value < 0.01) [41]. These results are in contrast to the findings of the other four included studies on malaria infections [41]. Three other included studies that presented age-disaggregated data identified children under the age of five as the most affected age group [42–44].

In addition, the included studies identified a positive association between the exposure to floods and the zoonotic disease taeniasis. Specifically, the presence of the antigen of taeniasis in the Kenyan study population increases when more grassland or agricultural land is flooded (OR = 1.09; 95% CI: 1.01-1.17, *p*-value = 0.03) [48].

### Evidence relevant to mechanisms of disease transmission

Our systematic review identified a number of mechanisms through which floods may contribute to disease transmission and negative health consequences.

First, floodwaters may damage or destroy buildings and infrastructure, which impedes access to healthcare and the implementation of preventative measures against disease transmission [42]. The loss of private dwellings in turn leads to overcrowding and displacement [39, 45]. Combined with the influx of aid workers, this may result in disease transmission to areas not previously affected [39]. Overcrowding and displacement exacerbates concerns about hygiene, water and adequate sanitation [39, 45]. Furthermore, it increases human-to-human interactions, which may increase disease transmission [39].

Second, floodwaters may overflow sanitation systems, causing contaminated environment and water sources [39]. This may hinder supply of or access to safe water sources, and can introduce infectious agents to areas which were previously not affected [39]. According to the same logic, floodwaters may spread diseases such as taeniasis by transporting the eggs (e.g., Taenia spp. eggs) to areas not previously affected [48].

Third, floods may create suitable breeding sites for disease vectors, causing the vector population to grow [42–44, 46, 47]. For example, stagnant water pools offer particularly favourable conditions for the Anopheles mosquito population, which in turn increases the risk of malaria transmission in affected areas [42, 43]. Additionally, floods contribute to high humidity or surface moisture, creating suitable conditions for the development and survival of disease vectors, larvae and eggs [42, 48].

### Critical appraisal of study quality

Due to the heterogeneity of the included study designs, no risk of bias assessment using a formal assessment tool was performed. Yet, in view of the substantial heterogeneity in included study designs, a critical assessment of study quality is all the more important to contextualize the results [50].

In the hierarchy of research designs, observational studies, which are inherently limited because exposure is not randomized, provide lower-quality evidence compared to randomized controlled trials [51]. In the case of the impact of flood exposure on health, observational studies offer evidence of critical importance, since it is ethically (and logistically) impossible to randomize people to potentially harmful exposures [52, 53].

It is, however, important to consider the limitations of observational studies. In general, non-randomized allocation to exposures poses several methodological issues for observational studies, including confounding, recall biases, indication and selection bias [54]. The principal constraint on observational study designs is that establishing cause-effect relations between exposures and health outcomes is very limited [55]. Due to non-randomization, observational designs cannot control for other unobservable factors or exposures that may cause or influence the result.

While all observational study designs can potentially include the above-mentioned disadvantages, different designs pose different shortcomings. Cross-sectional study designs, like those by Wardrop and colleagues (2015), Grossi-Soyster and colleagues (2017) and Mboera and colleagues (2011), cannot establish temporality (i.e. whether the health condition occurred prior to or in consequence of a flood) because information is gathered at a single point in time [38, 46, 48, 56]. In the case of Mboera and colleagues’ (2011) study, the authors adjusted for sex, age, anaemia and splenomegaly [38]. Other factors, however, such as the use of insecticide-treated bed nets, may have influenced the results. Despite the shortcomings of cross-sectional studies, they can be useful for determining whether an association exists in the first place and in situations in which reverse causality is unlikely.

Case-control study designs, as applied by Wardrop and colleagues (2013) and Sara and colleagues (2018), can suffer from selection, ascertainment and/or recall bias [45, 47]. Selection bias occurs because cases and controls are chosen after the development of a disease. If cases or controls die before the selection process, this may lead to a non-representative sample [57]. With regard to studies by Wardrop and colleagues (2013) and Sara and colleagues (2018), the results could have been affected by ascertainment bias, which can arise when there is more intense screening for a certain disease among individuals exposed to floods than among unexposed individuals [45, 47]. Wardrop and colleagues (2013) used frequency matching based on age group, gender and months of admission to the hospital to control for potential confounding [47]. A frequency matched case-control study matches controls with cases of a specific group to achieve a similar overall distribution [58]. The small sample size of the Sara and colleagues (2018) case-control study may have been more adequately addressed by employing a frequency matched case-control study [45, 57].

Boyce and colleagues (2016) used a difference-in-differences approach, which can be classified as a quasi-experimental study design [43]. The difference-in-difference method compares data from two groups over two time periods. In the Boyce and colleagues (2016) study, members of the treatment group were exposed to a flood-affected river because their village was next to the river, whereas the control group lived farther from the river [43]. Difference-in-difference designs allow for causal inference if the parallel trend assumption is fulfilled, which, in this case, requires that trends in malaria transmission were similar in exposed and unexposed villages in the period prior to the flooding. Boyce and colleagues (2016) provide compelling graphical evidence suggesting that the parallel trend assumption is fulfilled [43].

Since four of the included studies were partly based on survey data, they could have suffered from response biases [41, 45, 46, 48]. All studies collected health outcomes from laboratory diagnostics or through the assessment of a professional nurse, not through survey-based self-reports. Yet, when determining if an individual was exposed to floods, two studies relied on participants’ self-report [41, 45]. These results may thus be more prone to measurement error, for example due to recall bias.

## Discussion

The findings of the present review suggest that floods have a significant impact on different health outcomes in SSA. Only one of the included studies found that flood exposure reduces the likelihood of occurrence of a disease [41]. The remaining nine studies present evidence suggesting that flood exposure increases the probability of being affected by one of the identified health outcomes, including cholera, scabies, Rhodesian sleeping sickness, malaria, taeniasis and the occurrence of alphaviruses and flaviviruses.

The results of Rieckmann and colleagues’ (2018) study are supported by previous findings in the literature [39]. A study in Bangladesh found that the number of cholera cases was almost six times higher during a flood compared to the season-specific average [59].

Sara and colleagues’ (2018) findings are broadly supported by similar studies linking flood exposure with several infectious skin diseases [45, 60, 61]. In an urban community in central Vietnam, 69% of households experienced scabies infections after flood exposure [62].

A comparison of the findings of Grossi-Soyster and colleagues’ study to a recent study by Jesús Crespo and colleagues (2019), conducted in Puerto Rico, confirms that flood areas are positively correlated with dengue fever, a disease caused by a flavivirus [46, 63]. Looking at the adult population of the mosquito *Aedes albopictus*, the vector for chikungunya fever (a disease caused by an alphavirus), Roiz and colleagues (2015) observed explosive growth of this mosquito population after a flood [64].

Oyekale’s (2015) findings from a cross-sectional study in Cameroon not only contrast with the findings of our four other included studies on malaria infections, but also differ considerably from previous results in the literature [41]. In fact, a study that compared different ecosystems with the density of adult mosquito populations found that a significantly larger number of anopheline mosquitos, the mosquito species that transmits malaria, were collected from traditional flooding rice irrigation ecosystems than all other ecosystems [37].

The findings of the three studies that included children in their study population are in line with the Malaria World Report 2019, which also flagged infants and children under the age of five as high-risk groups for malaria infections [42–44, 65].

While it cannot be inferred that flood exposure affects the transmission of vector-borne disease more than water-borne or zoonotic diseases, our findings highlight that the transmission channels of vector-borne diseases are strongly affected by flood exposure. Overall, our study strengthens the assumption that floods create breeding habitats for insects, especially mosquitoes, which may result in population growth of disease vectors and a higher prevalence of vector-borne diseases in SSA. Further studies need to be carried out to validate if more breeding habitats result in a greater prevalence of vector-borne disease. With reference to Chirebvu and colleagues’ (2016) reasoning that transmission patterns of malaria may coincide with receding floods and not the onset of floods, it would be worth investigating the temporal relationship between the onset, peak and receding phase of floods and vector-borne disease [44].

The categorization by disease type highlighted existing knowledge gaps. Although the effects of natural disasters on mental health, such as post-traumatic stress disorder, anxiety and depression, are much discussed, we did not find any study that met the inclusion criteria and focused on mental health related outcomes [66, 67]. Most studies that covered mental health outcomes were excluded due to ineligible study designs during the study identification process. Despite a global increase in awareness of the importance of mental health, it has not been a prime focus in SSA given the dominant role played by other diseases [68]. Nonetheless, the burden of mental disorders and diseases is projected to increase in many sub-Saharan African countries [25].

Studies that concentrate on the effects of natural disasters on the nutritional state of individuals focus mostly on droughts and not on floods. However, floods can also endanger food supplies and food security, by damaging crops. In the worst case scenario, floods can lead to food crises, or even famine [69]. Exposure to famine has additional severe health consequences, such as lower growth and development among children [70]. Empirical studies have also shown that prenatal exposure to famine is associated with a higher risk of diabetes and schizophrenia, and lower adult height and poor cognitive development, which affects employment prospects and income earning opportunities in adulthood [71, 72]. This is particularly important for SSA, given that the region is home to 239 million people suffering from hunger, second only to Asia and the Pacific [73].

Our findings also highlight the need for more long-term evaluation of the impacts of flood exposure. Our systematic review did not distinguish between long- and short-term health effects because none of the studies discussed the long-term health effects of floods. Research has shown that there are long-term effects on health after exposure to floods. For example, chronic disease and related conditions can worsen after exposure to natural disasters [51]. Evidence from other parts of the world shows that birth outcomes and pregnancy may be affected by flood exposure. Therefore the long-term impact evaluations of flood exposure should cover the health effects on birth outcomes and pregnancy in addition to non-communicable diseases, nutrition and mental health [74, 75]. Given that several studies suggest that women are more affected in the disaster period than men, research should also focus on the health effects of flood exposure on women specifically [76, 77]. In particular, there is a lack of understanding and quantitative research into the risk of domestic violence and disrupted access to sexual and reproductive health care services after flood impact [78, 79].

The findings of this systematic review highlight that the peak of malaria cases coincides with receding floods, several weeks after the initial onset of the flood [42–44]. This time frame may provide an important opportunity for targeted vector-control interventions to mitigate malaria epidemics in the post flood period. It also shows that interventions must be sustained over a longer period of time after the initial flood event [43]. Since floods often occur unexpectedly, the implementation of preventive measures in flood-prone regions, including the distribution of insecticide-treated bed nets, should be a key policy priority. Supplying safe drinking water and providing sufficient emergency shelter in disaster areas are other important interventions to mitigate the risk of disease outbreaks in the aftermath of floods.

It is plausible that a number of limitations have influenced the results obtained. The results were characterised by a high level of heterogeneity. In total, six different health outcomes were reported, of which five were examined by only one study each. Heterogeneity was also present with regards to the geographic context of each individual study. The systematic review includes evidence from seven different countries (excluding the Rieckmann and colleagues (2018) study [39]). It is therefore possible that differences in the findings are driven by differing climatic and endemic disease conditions in the study country.

The results are based on a small number of included studies, which means they cannot be generalized and suggests general neglect of the study area.

This systematic review focused on quantitative evidence and excluded qualitative evidence. Therefore, a possible and expected shortcoming is a lack of understanding regarding the mechanisms behind the effects of floods on human health.

In addition, this systematic review is prone to different types of reporting biases, such as publication and language bias. Since only English-language reports were included in this systematic review, valuable studies published in other languages were automatically excluded. Furthermore, this systematic review presents only published studies, which can cause publication bias, because studies with statistically significant results are more likely to be published than studies with results that are not statistically significant [80, 81].

Lastly, no systematic risk of bias assessment was conducted in this systematic review. To the best of our knowledge, there is no available risk of bias assessment tool that can encompass case-control studies, cross-sectional studies, and quasi-experimental designs.

## Conclusion

Our review has identified ten studies, of which nine point to an increased risk of infection with cholera, scabies, taeniasis, Rhodesian sleeping sickness, malaria, alphaviruses and flaviviruses following floods. Just one of the ten included studies suggests a decreased risk of infection with malaria after flood exposure.

This systematic review provides deeper insights into the complex relationship between flood exposure and health outcomes. Our results should motivate further research in the field because they highlight crucial knowledge gaps. This research should focus on non-communicable diseases, nutrition, mental health, pregnancy, gender, and birth outcomes. Considerably more work is needed to determine the long-term health effects of floods. Furthermore, we are still not able to explain most of the specific mechanisms behind flood-related health risks.

This is the first comprehensive assessment of flood effects on human health in SSA and adds to the growing body of research into the many pathways by which floods affect humans. Climate change means that policymakers in SSA must act to protect the health of their populations based on a limited, but growing, body of evidence. Despite increasing efforts to develop policies and strategies to reduce disaster risk, most sub-Saharan African countries’ disaster preparedness is not well established [82]. Africa’s low contribution, but high vulnerability to climate change, calls for global cooperation and an effective international response [83].

## Supplementary Information

**Additional File 1.** Search strategy.

**Additional File 2.** PRISMA Checklist.

## Data Availability

All data generated or analysed during this study are included in this published article and its supplementary information files.

## Abbreviations

SSA: Sub-Saharan Africa
EM-DAT: Emergency Events Database
IRR: Incidence rate ratio
CI: Confidence interval
ME: Marginal effect
OR: Odds ratio
SPR: Slide positivity rate
ARR: Adjusted rate ratio
ρ: Pearson correlation coefficient
aOR: Adjusted odds ratio

## Footnotes

1 The Slide Positivity Rate is defined as the number of laboratory-confirmed malaria cases per 100 suspected cases [49].

2 It must be noted that the Pearson’s rho for flood discharge reported by Chirebvu and colleagues (2016) deviates between the table (ρ = 0.396) and the manuscript (ρ = 0.497).
