# Supplementary material for: Epidemiology of floods in sub-Saharan Africa: a systematic review of health outcomes
Source: BMC Public Health. 2022 Feb 10;22:268. doi: 10.1186/s12889-022-12584-4 (PMC8830087; doi:10.1186/s12889-022-12584-4)
Supplement: Supplementary file 1 — Additional File 1. Search strategy. [file 12889_2022_12584_MOESM1_ESM.pdf]

Additional File 1. Search strategy

1. Pubmed

Date	17.09.2020
Search Number	String
#1	(flood* OR hydrological event* OR deluge* OR torrent* OR “high water” OR “high tide” OR stormwater* OR waterlogging OR “water logging” OR storm surge* OR inundation*) AND
#2	(health OR morbidit* OR mortalit* OR death* OR sick* OR illn* OR wound* OR injur* OR accident* OR disease* OR disorder* OR syndrome* OR mental OR physical OR emotional OR suicide* OR infection* OR abnormalit* OR pregnanc* OR complication* OR genetic* OR trauma* OR nervous system* OR shock* OR drown* OR starv* OR neoplasm* OR cancer* OR hypersensivit* OR condition* OR sign* OR symptom* OR finding* OR sleep OR syndrome* OR stress* OR diet* OR diagnos* OR epidemi* OR virus OR phsycholog* OR immun* OR nutrition* OR fever* OR water-related OR water related OR water-borne OR water borne OR bite* OR side effect* OR risk factor* OR outbreak* OR mosquito* OR malaria OR diarrhea OR diarrhoea OR anxiet* OR cholera) AND
#3	("SSA" OR Africa OR "Sub-Saharan Africa" OR "Subsaharan Africa" OR "Africa, Sub-Saharan" OR "Africa South of the Sahara" OR Subsaharan OR Sub-Saharan OR "Central Africa" OR "Africa, Central" OR Cameroon* OR "United Republic of Cameroon" OR "Central African Republic" OR "Ubangi-Shari" OR Chad OR Congo OR "Republic of the Congo" OR Brazzaville OR "Democratic Republic of the Congo" OR Kinshasa OR Zaire OR "Belgian Congo" OR Katanga OR "Republic of Equatorial Guinea" OR "Spanish Guinea" OR "Guinea, Spanish" OR "Rio Muni" OR "Equatorial Guinea" OR "Gabonese Republic" OR Gabon OR "Sao Tome and Principe" OR "East Africa" OR "Eastern Africa" OR "British Indian Ocean Territory" OR "Africa, Eastern" OR "Republic of Burundi" OR Urundi OR Burundi OR Djibouti OR "Republic of Djibouti" OR "French Somaliland" OR Eritrea OR "Federal Democratic Republic of Ethiopia" OR Ethiopia OR Kenya OR "Republic of Kenya" OR "Republic of Rwanda" OR Ruanda OR Rwanda OR Somalia OR "South Sudan" OR "Republic of the Sudan" OR Sudan OR "United Republic of Tanzania" OR Zanzibar OR Tanganyika OR Tanzania OR "Republic of Uganda" OR Uganda OR "Africa, Southern" OR "Southern Africa" OR Angola OR Bechuanaland OR Kalahari OR Botswana OR Eswatini OR Swaziland OR Basutoland OR "Kingdom of Lesotho" OR Lesotho OR "Republic of Malawi" OR Nyasaland OR Malawi OR "Republic of Mozambique" OR "Portuguese East Africa" OR Mozambique OR "Southwest Africa" OR "Republic of Namibia" OR "South West Africa" OR Namibia OR "Union of South Africa" OR "Republic of South Africa" OR "South Africa" OR "Northern Rhodesia" OR "Republic of Zambia" OR Zambia OR

	"Zimbabwe Rhodesia" OR "Southern Rhodesia" OR "Republic of Zimbabwe" OR "Rhodesia, Southern" OR Zimbabwe OR "Africa, West" OR "West Africa" OR "Western Africa" OR "Africa, Western" OR "Republic of Benin" OR Benin OR Dahomey OR "Upper Volta" OR "Burkina Fasso" OR "Burkina Faso" OR "Republic of Cape Verde" OR "Cape Verde" OR "Cabo Verde" OR "Ivory Coast" OR "Cote d'Ivoire" OR "Republic of the Gambia" OR "Gambia" OR "Republic of Ghana" OR "Gold Coast" OR Ghana OR "Guinea, French" OR "Republic of Guinea" OR "French Guinea" OR Guinea OR "Republic of Guinea-Bissau" OR "Portuguese Guinea" OR "Guinea-Bissau, Republic of" OR "Guinea-Bissau" OR "Republic of Liberia" OR Liberia OR "Republic of Mali" OR Mali OR Mauritania OR "Republic of Niger" OR Niger OR "Federal Republic of Nigeria" OR Nigeria OR "Republic of Senegal" OR Senegal OR "Republic of Sierra Leone" OR "Sierra Leone" OR "Togolese Republic" OR Togo)
#4	Search #1 & #2 & #3
Timespan	2010 - 2020
Studies found	1649

2. Web of Science
    a. Web of Science Core Collection

Date	17.09.2020
Search Number	String
#1	TOPIC: (flood* OR hydrological event* OR deluge* OR torrent* OR “high water” OR “high tide” OR stormwater* OR waterlogging OR “water logging” OR storm surge* OR inundation*) AND
#2	TITLE: (health OR morbidit* OR mortalit* OR death* OR sick* OR illn* OR wound* OR injur* OR accident* OR disease* OR disorder* OR syndrome* OR mental OR physical OR emotional OR suicide* OR infection* OR abnormalit* OR pregnanc* OR complication* OR genetic* OR trauma* OR nervous system* OR shock* OR drown* OR starv* OR neoplasm* OR cancer* OR hypersensivit* OR condition* OR sign* OR symptom* OR finding* OR sleep OR syndrome* OR stress* OR diet* OR diagnos* OR epidemi* OR virus OR phsycholog* OR immun* OR nutrition* OR fever* OR water-related OR water related OR water-borne OR water borne OR bite* OR side effect* OR risk factor* OR outbreak* OR mosquito* OR malaria OR diarrhea OR diarrhoea OR anxiet* OR cholera) AND
#3	TOPIC: ("SSA" OR Africa OR "Sub-Saharan Africa" OR "Subsaharan Africa" OR "Africa, Sub-Saharan" OR "Africa South of the Sahara" OR Subsaharan OR Sub-Saharan OR "Central Africa" OR "Africa, Central" OR Cameroon* OR "United Republic of Cameroon" OR "Central African Republic" OR "Ubangi-Shari" OR Chad OR Congo OR "Republic of the Congo" OR Brazzaville OR "Democratic Republic of the Congo" OR Kinshasa OR Zaire OR "Belgian Congo" OR

	Katanga OR "Republic of Equatorial Guinea" OR "Spanish Guinea" OR "Guinea, Spanish" OR "Rio Muni" OR "Equatorial Guinea" OR "Gabonese Republic" OR Gabon OR "Sao Tome and Principe" OR "East Africa" OR "Eastern Africa" OR "British Indian Ocean Territory" OR "Africa, Eastern" OR "Republic of Burundi" OR Urundi OR Burundi OR Djibouti OR "Republic of Djibouti" OR "French Somaliland" OR Eritrea OR "Federal Democratic Republic of Ethiopia" OR Ethiopia OR Kenya OR "Republic of Kenya" OR "Republic of Rwanda" OR Ruanda OR Rwanda OR Somalia OR "South Sudan" OR "Republic of the Sudan" OR Sudan OR "United Republic of Tanzania" OR Zanzibar OR Tanganyika OR Tanzania OR "Republic of Uganda" OR Uganda OR "Africa, Southern" OR "Southern Africa" OR Angola OR Bechuanaland OR Kalahari OR Botswana OR Eswatini OR Swaziland OR Basutoland OR "Kingdom of Lesotho" OR Lesotho OR "Republic of Malawi" OR Nyasaland OR Malawi OR "Republic of Mozambique" OR "Portuguese East Africa" OR Mozambique OR "Southwest Africa" OR "Republic of Namibia" OR "South West Africa" OR Namibia OR "Union of South Africa" OR "Republic of South Africa" OR "South Africa" OR "Northern Rhodesia" OR "Republic of Zambia" OR Zambia OR "Zimbabwe Rhodesia" OR "Southern Rhodesia" OR "Republic of Zimbabwe" OR "Rhodesia, Southern" OR Zimbabwe OR "Africa, West" OR "West Africa" OR "Western Africa" OR "Africa, Western" OR "Republic of Benin" OR Benin OR Dahomey OR "Upper Volta" OR "Burkina Fasso" OR "Burkina Faso" OR "Republic of Cape Verde" OR "Cape Verde" OR "Cabo Verde" OR "Ivory Coast" OR "Cote d'Ivoire" OR "Republic of the Gambia" OR "Gambia" OR "Republic of Ghana" OR "Gold Coast" OR Ghana OR "Guinea, French" OR "Republic of Guinea" OR "French Guinea" OR Guinea OR "Republic of Guinea-Bissau" OR "Portuguese Guinea" OR "Guinea-Bissau, Republic of" OR "Guinea-Bissau" OR "Republic of Liberia" OR Liberia OR "Republic of Mali" OR Mali OR Mauritania OR "Republic of Niger" OR Niger OR "Federal Republic of Nigeria" OR Nigeria OR "Republic of Senegal" OR Senegal OR "Republic of Sierra Leone" OR "Sierra Leone" OR "Togolese Republic" OR Togo)
#4	Search #1 & #2 & #3
Timespan	2010 - 2020
Studies found	348

b. SciELO Citation Index

Date	17.09.2020
Search Number	String
#1	TOPIC: (flood* OR hydrological event* OR deluge* OR torrent* OR “high water” OR “high tide” OR stormwater* OR waterlogging OR “water logging” OR storm surge* OR inundation*) AND

#2	TOPIC: (health OR morbidit* OR mortalit* OR death* OR sick* OR illn* OR wound* OR injur* OR accident* OR disease* OR disorder* OR syndrome* OR mental OR physical OR emotional OR suicide* OR infection* OR abnormalit* OR pregnanc* OR complication* OR genetic* OR trauma* OR nervous system* OR shock* OR drown* OR starv* OR neoplasm* OR cancer* OR hypersensivit* OR condition* OR sign* OR symptom* OR finding* OR sleep OR syndrome* OR stress* OR diet* OR diagnos* OR epidemi* OR virus OR phsycholog* OR immun* OR nutrition* OR fever* OR water-related OR water related OR water-borne OR water borne OR bite* OR side effect* OR risk factor* OR outbreak* OR mosquito* OR malaria OR diarrhea OR diarrhoea OR anxiet* OR cholera) AND
#3	("SSA" OR Africa OR "Sub-Saharan Africa" OR "Subsaharan Africa" OR "Africa, Sub-Saharan" OR "Africa South of the Sahara" OR Subsaharan OR Sub-Saharan OR "Central Africa" OR "Africa, Central" OR Cameroon* OR "United Republic of Cameroon" OR "Central African Republic" OR "Ubangi-Shari" OR Chad OR Congo OR "Republic of the Congo" OR Brazzaville OR "Democratic Republic of the Congo" OR Kinshasa OR Zaire OR "Belgian Congo" OR Katanga OR "Republic of Equatorial Guinea" OR "Spanish Guinea" OR "Guinea, Spanish" OR "Rio Muni" OR "Equatorial Guinea" OR "Gabonese Republic" OR Gabon OR "Sao Tome and Principe" OR "East Africa" OR "Eastern Africa" OR "British Indian Ocean Territory" OR "Africa, Eastern" OR "Republic of Burundi" OR Urundi OR Burundi OR Djibouti OR "Republic of Djibouti" OR "French Somaliland" OR Eritrea OR "Federal Democratic Republic of Ethiopia" OR Ethiopia OR Kenya OR "Republic of Kenya" OR "Republic of Rwanda" OR Ruanda OR Rwanda OR Somalia OR "South Sudan" OR "Republic of the Sudan" OR Sudan OR "United Republic of Tanzania" OR Zanzibar OR Tanganyika OR Tanzania OR "Republic of Uganda" OR Uganda OR "Africa, Southern" OR "Southern Africa" OR Angola OR Bechuanaland OR Kalahari OR Botswana OR Eswatini OR Swaziland OR Basutoland OR "Kingdom of Lesotho" OR Lesotho OR "Republic of Malawi" OR Nyasaland OR Malawi OR "Republic of Mozambique" OR "Portuguese East Africa" OR Mozambique OR "Southwest Africa" OR "Republic of Namibia" OR "South West Africa" OR Namibia OR "Union of South Africa" OR "Republic of South Africa" OR "South Africa" OR "Northern Rhodesia" OR "Republic of Zambia" OR Zambia OR "Zimbabwe Rhodesia" OR "Southern Rhodesia" OR "Republic of Zimbabwe" OR "Rhodesia, Southern" OR Zimbabwe OR "Africa, West" OR "West Africa" OR "Western Africa" OR "Africa, Western" OR "Republic of Benin" OR Benin OR Dahomey OR "Upper Volta" OR "Burkina Fasso" OR "Burkina Faso" OR "Republic of Cape Verde" OR "Cape Verde" OR "Cabo Verde" OR "Ivory Coast" OR "Cote d'Ivoire" OR "Republic of the Gambia" OR "Gambia" OR "Republic of Ghana" OR "Gold Coast" OR

	Ghana OR "Guinea, French" OR "Republic of Guinea" OR "French Guinea" OR Guinea OR "Republic of Guinea-Bissau" OR "Portuguese Guinea" OR "Guinea-Bissau, Republic of" OR "Guinea-Bissau" OR "Republic of Liberia" OR Liberia OR "Republic of Mali" OR Mali OR Mauritania OR "Republic of Niger" OR Niger OR "Federal Republic of Nigeria" OR Nigeria OR "Republic of Senegal" OR Senegal OR "Republic of Sierra Leone" OR "Sierra Leone" OR "Togolese Republic" OR Togo)
#4	Search #1 & #2 & #3
Timespan	2010 - 2020
Studies found	104

c. Medline

Date	17.09.2020
Search Number	String
#1	TOPIC: (flood* OR hydrological event* OR deluge* OR torrent* OR "high water" OR "high tide" OR stormwater* OR waterlogging OR "water logging" OR storm surge* OR inundation*) AND
#2	TITLE: (health OR morbidit* OR mortalit* OR death* OR sick* OR illn* OR wound* OR injur* OR accident* OR disease* OR disorder* OR syndrome* OR mental OR physical OR emotional OR suicide* OR infection* OR abnormalit* OR pregnanc* OR complication* OR genetic* OR trauma* OR nervous system* OR shock* OR drown* OR starv* OR neoplasm* OR cancer* OR hypersensivit* OR condition* OR sign* OR symptom* OR finding* OR sleep OR syndrome* OR stress* OR diet* OR diagnos* OR epidemi* OR virus OR phsycholog* OR immun* OR nutrition* OR fever* OR water-related OR water related OR water-borne OR water borne OR bite* OR side effect* OR risk factor* OR outbreak* OR mosquito* OR malaria OR diarrhea OR diarrhoea OR anxiet* OR cholera) AND
#3	TOPIC: ("SSA" OR Africa OR "Sub-Saharan Africa" OR "Subsaharan Africa" OR "Africa, Sub-Saharan" OR "Africa South of the Sahara" OR Subsaharan OR Sub-Saharan OR "Central Africa" OR "Africa, Central" OR Cameroon* OR "United Republic of Cameroon" OR "Central African Republic" OR "Ubangi-Shari" OR Chad OR Congo OR "Republic of the Congo" OR Brazzaville OR "Democratic Republic of the Congo" OR Kinshasa OR Zaire OR "Belgian Congo" OR Katanga OR "Republic of Equatorial Guinea" OR "Spanish Guinea" OR "Guinea, Spanish" OR "Rio Muni" OR "Equatorial Guinea" OR "Gabonese Republic" OR Gabon OR "Sao Tome and Principe" OR "East Africa" OR "Eastern Africa" OR "British Indian Ocean Territory" OR "Africa, Eastern" OR "Republic of Burundi" OR Urundi OR Burundi OR Djibouti OR "Republic of Djibouti" OR "French Somaliland" OR Eritrea OR "Federal Democratic Republic of Ethiopia" OR Ethiopia OR Kenya OR "Republic of Kenya"

	OR "Republic of Rwanda" OR Ruanda OR Rwanda OR Somalia OR "South Sudan" OR "Republic of the Sudan" OR Sudan OR "United Republic of Tanzania" OR Zanzibar OR Tanganyika OR Tanzania OR "Republic of Uganda" OR Uganda OR "Africa, Southern" OR "Southern Africa" OR Angola OR Bechuanaland OR Kalahari OR Botswana OR Eswatini OR Swaziland OR Basutoland OR "Kingdom of Lesotho" OR Lesotho OR "Republic of Malawi" OR Nyasaland OR Malawi OR "Republic of Mozambique" OR "Portuguese East Africa" OR Mozambique OR "Southwest Africa" OR "Republic of Namibia" OR "South West Africa" OR Namibia OR "Union of South Africa" OR "Republic of South Africa" OR "South Africa" OR "Northern Rhodesia" OR "Republic of Zambia" OR Zambia OR "Zimbabwe Rhodesia" OR "Southern Rhodesia" OR "Republic of Zimbabwe" OR "Rhodesia, Southern" OR Zimbabwe OR "Africa, West" OR "West Africa" OR "Western Africa" OR "Africa, Western" OR "Republic of Benin" OR Benin OR Dahomey OR "Upper Volta" OR "Burkina Fasso" OR "Burkina Faso" OR "Republic of Cape Verde" OR "Cape Verde" OR "Cabo Verde" OR "Ivory Coast" OR "Cote d'Ivoire" OR "Republic of the Gambia" OR "Gambia" OR "Republic of Ghana" OR "Gold Coast" OR Ghana OR "Guinea, French" OR "Republic of Guinea" OR "French Guinea" OR Guinea OR "Republic of Guinea-Bissau" OR "Portuguese Guinea" OR "Guinea-Bissau, Republic of" OR "Guinea-Bissau" OR "Republic of Liberia" OR Liberia OR "Republic of Mali" OR Mali OR Mauritania OR "Republic of Niger" OR Niger OR "Federal Republic of Nigeria" OR Nigeria OR "Republic of Senegal" OR Senegal OR "Republic of Sierra Leone" OR "Sierra Leone" OR "Togolese Republic" OR Togo)
#4	Search #1 & #2 & #3
Timespan	2010 - 2020
Studies found	199
